# Design and validation of a 63K genome-wide SNP-genotyping platform for caribou/reindeer (*Rangifer tarandus*)

**DOI:** 10.1186/s12864-022-08899-6

**Published:** 2022-10-05

**Authors:** Alexandra Carrier, Julien Prunier, William Poisson, Mallorie Trottier-Lavoie, Isabelle Gilbert, Maria Cavedon, Kisun Pokharel, Juha Kantanen, Marco Musiani, Steeve D. Côté, Vicky Albert, Joëlle Taillon, Vincent Bourret, Arnaud Droit, Claude Robert

**Affiliations:** 1 Département de sciences animales, Faculté de l’agriculture et d’alimentation, Université Laval, Quebec City, Québec Canada; 2 Centre de recherche en reproduction, développement et santé intergénérationnelle (CRDSI), Quebec City, Québec Canada; 3 Réseau Québécois en reproduction (RQR), Saint-Hyacinthe, Québec Canada; 4 Département de médecine moléculaire, Faculté de médecine, Université Laval, Quebec City, Québec Canada; 5 Department of biological sciences, Faculty of Science, University of Calgary, Calgary, Canada; 6 Natural Resources Institute Finland, Jokioinen, Finland; 7 Département de biologie - Faculté de sciences et génie, Caribou Ungava, Université Laval, Quebec City, Québec Canada; 8 Ministère des Forêts, de la Faune et des Parcs du Québec (MFFP), Quebec City, Québec Canada; 9 Department of Biological, Geological and Environmental Sciences (BiGeA), University of Bologna, Bologna, Italy

**Keywords:** SNP chip, *Rangifer tarandus*, Genotyping, Next generation sequencing, Genomics

## Abstract

**Background:**

Development of large single nucleotide polymorphism (SNP) arrays can make genomic data promptly available for conservation problematic. Medium and high-density panels can be designed with sufficient coverage to offer a genome-wide perspective and the generated genotypes can be used to assess different genetic metrics related to population structure, relatedness, or inbreeding. SNP genotyping could also permit sexing samples with unknown associated metadata as it is often the case when using non-invasive sampling methods favored for endangered species. Genome sequencing of wild species provides the necessary information to design such SNP arrays. We report here the development of a SNP-array for endangered *Rangifer tarandus* using a multi-platform sequencing approach from animals found in diverse populations representing the entire circumpolar distribution of the species.

**Results:**

From a very large comprehensive catalog of SNPs detected over the entire sample set (N = 894), a total of 63,336 SNPs were selected. SNP selection accounted for SNPs evenly distributed across the entire genome (~ every 50Kb) with known minor alleles across populations world-wide. In addition, a subset of SNPs was selected to represent rare and local alleles found in Eastern Canada which could be used for ecotype and population assignments - information urgently needed for conservation planning. In addition, heterozygosity from SNPs located in the X-chromosome and genotyping call-rate of SNPs located into the SRY gene of the Y-chromosome yielded an accurate and robust sexing assessment. All SNPs were validated using a high-throughput SNP-genotyping chip.

**Conclusion:**

This design is now integrated into the first genome-wide commercially available genotyping platform for *Rangifer tarandus*. This platform would pave the way to future genomic investigation of populations for this endangered species, including estimation of genetic diversity parameters, population assignments, as well as animal sexing from genetic SNP data for non-invasive samples.

**Supplementary Information:**

The online version contains supplementary material available at 10.1186/s12864-022-08899-6.

## Background

For decades, population genetics approaches have been deployed to inform species conservation efforts [1]. Molecular markers such as mitochondrial DNA sequences and microsatellites have been used to answer conservation genetic questions such as estimating relatedness between individuals [2, 3] or effective population size [4, 5], and ascertaining population structure to delineate conservation units [6, 7]. In the past decades, genetic studies have also started to rely on the analysis of single nucleotide polymorphisms (SNPs), which are highly abundant across genomes (i.e. genome-wide) and efficiently genotyped using high-throughput genotyping assays.

Commercial genotyping platforms have been designed in human and livestock following genome sequencing and assembly of draft reference genomes, which enabled comparative genomics approaches to identify potent loci involved in diseases and zootechnic performances [8–11], for instance. This genome-wide presence of genotyped SNPs provides advantages over other molecular markers by offering a broader view of the individual’s genetic constitution. For example, Tokarska et al. [12] showed that a low-cost panel of 50 highly informative SNPs were better suited than 17 microsatellites to conduct paternity and identity analyses in European Bison (B*ison bonasus*). Furthermore, SNPs genotyped in high numbers (e.g. order of thousands) for multiple individuals improve the precision and accuracy of the parameters estimated in genomic population analyses [13–15]. For instance, Barbosa et al. [16] demonstrated that higher/deeper levels of delineation of conservation units in populations of Cabrera voles (*Microtus cabrerae*) could be obtained from SNPs data in comparison to previous mitochondrial DNA markers [16]. Thus, natural populations management tools can highly benefit from the development of SNP arrays.

*Rangifer tarandus*, commonly named caribou in North America and reindeer in Eurasia, is an Arctic emblematic species due to its wide range over the entire circumpolar region. Rangifer populations are commonly distinguished and grouped using the concept of ecotype, which is attributed to a population or group of populations presenting particular life history traits, ecological preferences, or different behaviours. In North America, for instance, four main caribou ecotypes are found: arctic (Peary) caribou, boreal caribou (sedentary in the boreal forest), mountain caribou, and migratory caribou [17]. Ecotypes can be managed differently, and caribou ecotypes have different legal protection status in Canada (COSEWiC 2014, 2017). However, all caribou ecotypes and several populations are currently drawing much attention due to important and rapid population declines. Previous studies have used microsatellites and mitochondrial DNA to investigate population structure and gene flow, and discriminate ecotypes – all essential aspects to promote proper population management [18]. However, recent draft genome assemblies have been reported [19–21] and have paved the way for the development of genomic tools such as a SNP array that would enable more efficient and valuable conservation tools needed for all *Rangifer* populations and ecotypes.

Herein, we report the development of a genome-wide panel of SNPs for *Rangifer tarandus* integrated in the design of a high-throughput SNP-genotyping chip based on the Illumina platform. Whole genome sequencing and genotyping-by-sequencing approaches were used to obtain a comprehensive list of SNPs found in numerous populations covering the circumpolar distribution. Subpanels of ecotype- and population-specific alleles found in the province of Quebec (Canada) and another subpanel of behaviour-associated SNPs previously described by Cavedon et al. [22] were also included. Development and efficiency of this novel genotyping platform for *Rangifer tarandus* are described.

## Results

### Sequencing data and SNP detection

**Fig. 1:**
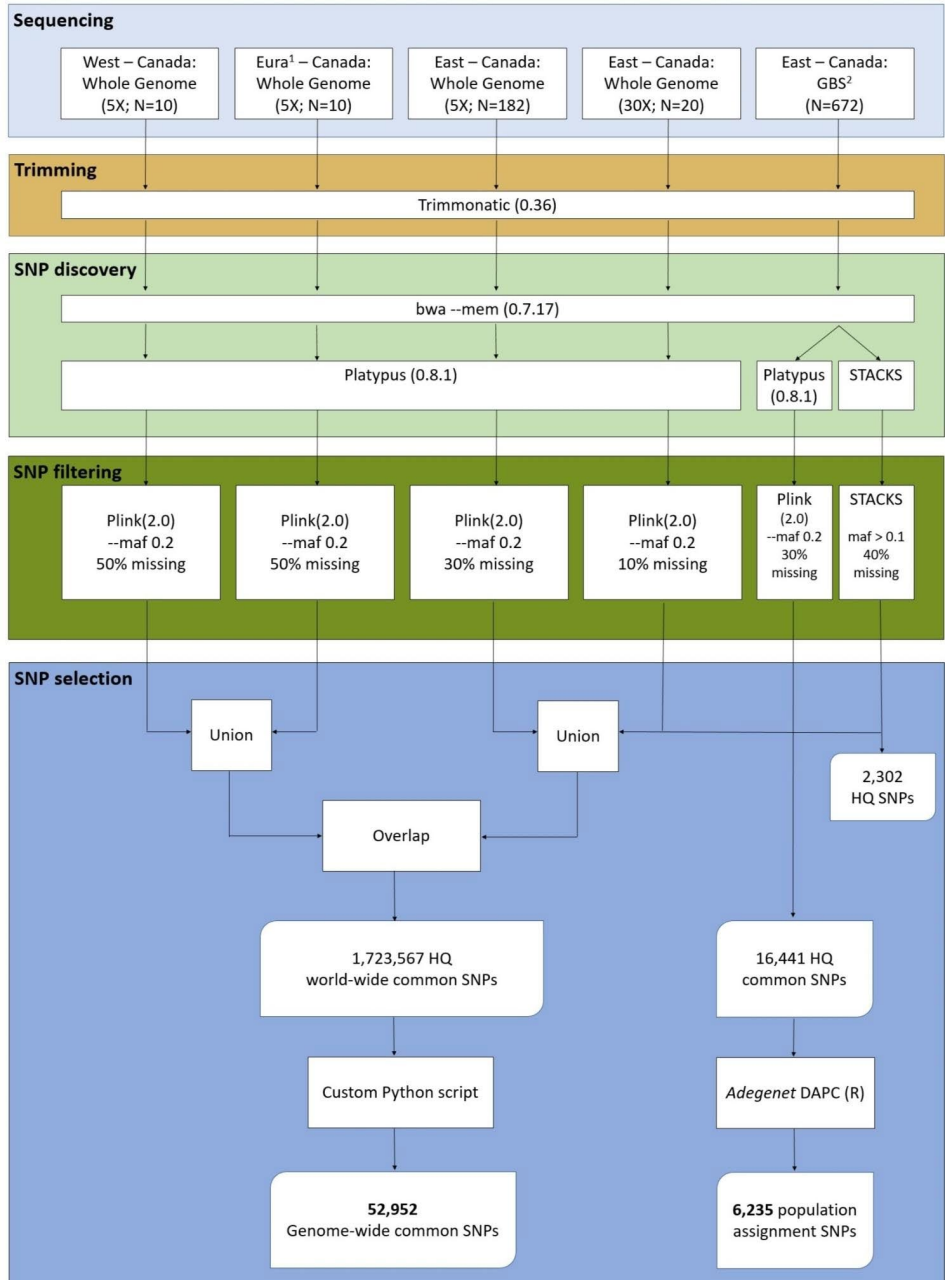
SNP discovery flowchart; including sampling, sequencing technologies, computer programs, and filtering criteria used for SNP discovery and integration into the genotyping array. ^1^Eura: Eurasian; ^2^GBS: Genotyping-By-Sequencing; maf: minor allele frequency; HQ: high-quality

Two sequencing strategies were deployed to maximize the breadth and depth of the genomic survey to generate a list of SNPs to be targeted by the genotyping platform. While whole-genome sequencing (WGS) allows to detect every polymorphism within and between individuals, provided a sufficient sequencing effort, cost is still prohibitive making it less suitable for in depth coverage of large cohorts of animals. On the other hand, genotyping-by-sequencing (GBS) is a cost-effective approach due to its reduced genomic representation therefore allowing to survey a fraction of the genome (1–3%) with good depth for a large number of samples. Both methods are complementary and were used to sequence five different sample sets (Table 1) that were later analyzed using different parameters in accordance with sample size, origin, and specific objectives (population assignment or homogeneous genome distribution; Fig. 1).

**Table 1:** Sequencing approaches and SNP yields in this study

Description	WGS 30X - EastCan	WGS 5X - EastCan	WGS 5X - West Can	WGS 5X - EUR	GBS-QC
Number of samples	20	182	10	10	672
Tissues	Ear punches	Ear punches and hairs	Ear punches	Ear punches and hairs	Ear punches and hairs
Samples population	N-E Canada	N-E Canada	N-W Canada	Eurasia	N-E Canada
Libraries	Shotgun DNA library	Shotgun DNA library	Shotgun DNA library	Shotgun DNA library	Two-enzyme protocol (Pst1/Msp1)
Illumina sequencer	HiSeqX	NovaSeq 6000	HiSeq 4000	NovaSeq 6000	NovaSeq 6000
Average number of reads per sample (M)	479.9	43.8	60.1	47.0	3.7
Read length	PE 150	PE 150	PE 150	PE150	PE 150
N raw SNPs	22914.4k	29826.9k	6119.8k	14675.6k	1061.5k
N high quality SNPs	6748k	139.4k	177.7k	3568.3k	16.4k

Illumina sequencing is well-known for its high sequencing quality as it was the case for all datasets presented in this work (Table 1) with an average Phred quality score of 35.6 for both low coverage WGS and GBS data (the average sequencing quality for previously published high coverage WGS being 37.7;[21]). The low coverage WGS generated an average number of 89.6 million reads per sample while 76.73 million reads aligned to the reference genome, which represented an average coverage of 4.5X. The GBS sequencing approach generated an average number of 4.27 million reads per sample, which translated into an average coverage of 5.75X over all regions targeted using the restriction enzymes.

**Fig. 2:**
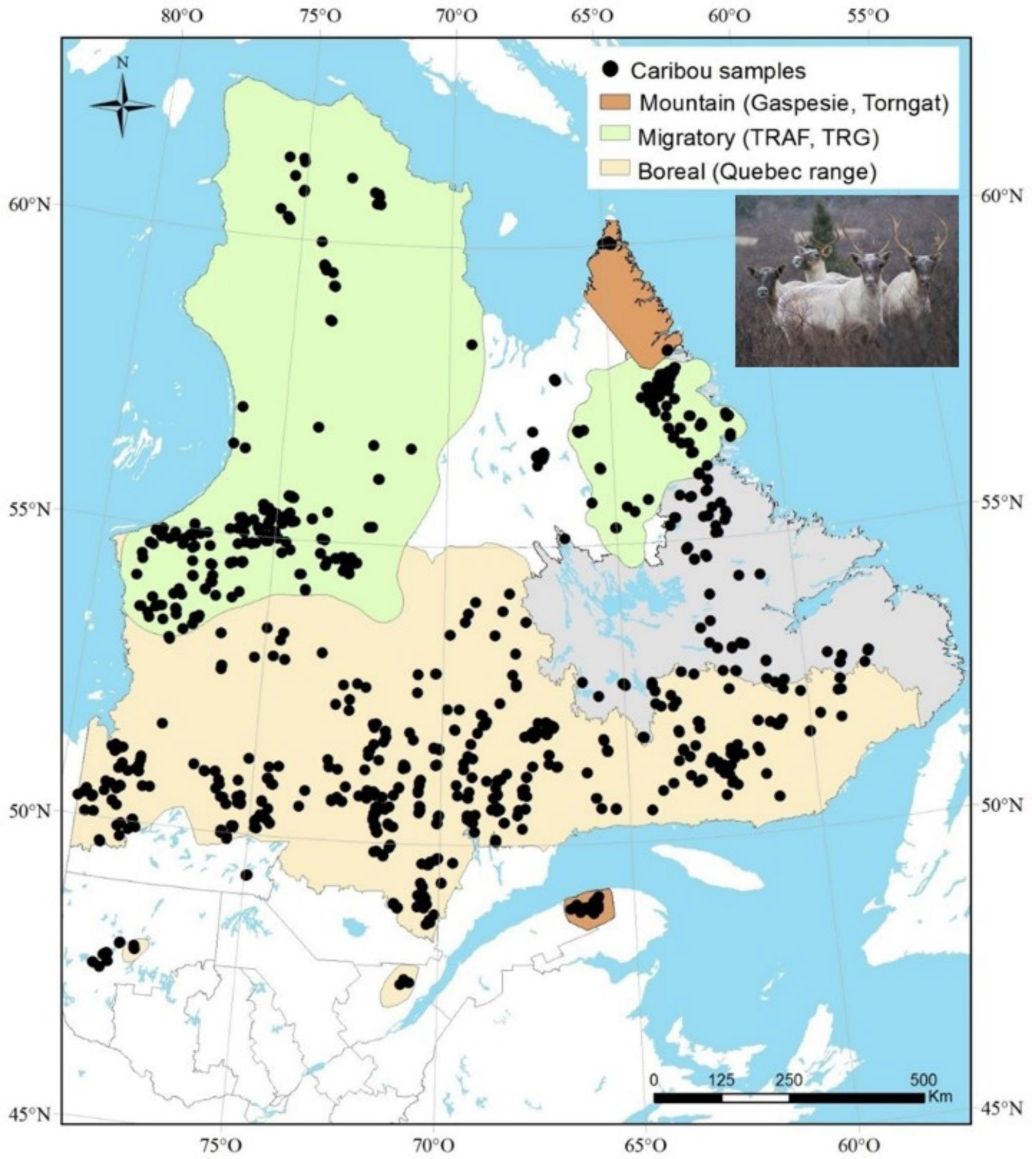
Geographic distribution of Caribou sampled in the East Canada. Ranges for the migratory ecotype are based on recent data (2019) whereas Mountain and Boreal ranges are based on data collected from previous years. The assignment of individuals to an ecotype was ensured by telemetry monitoring. TRAF: “Rivière-aux-feuilles” herd. TRG: “Rivière-Georges” herd. Photo credit: Joëlle Taillon

From alignments of this 5X sequencing data with the most complete genome assembly available for this species in the NCBI ([21]; GCA_019903745.1), the platypus SNP caller detected a total of 29,826,954 SNPs for the large sample set from East Canada (Figs. 2), 6,119,830 SNPs for the West-Canadian group and 14,675,605 for the Eurasian group (Table 1).

After filtering for minimal MAF and maximal proportion of missing genotypes for one SNP (Figs. 1), 139,381 (0.47%) SNPs were remaining for the East-Canadian group, 177,686 SNPs (2.9%) for the West-Canadian group and 3,568,320 (24.3%) SNPs for the Eurasian group (Table 1). Using the high coverage WGS including 20 individuals, the platypus SNP caller detected a total of 22,914,427 SNPs which yielded 6,747,954 (29.4%) remaining SNPs after filtering.

**Fig. 3:**
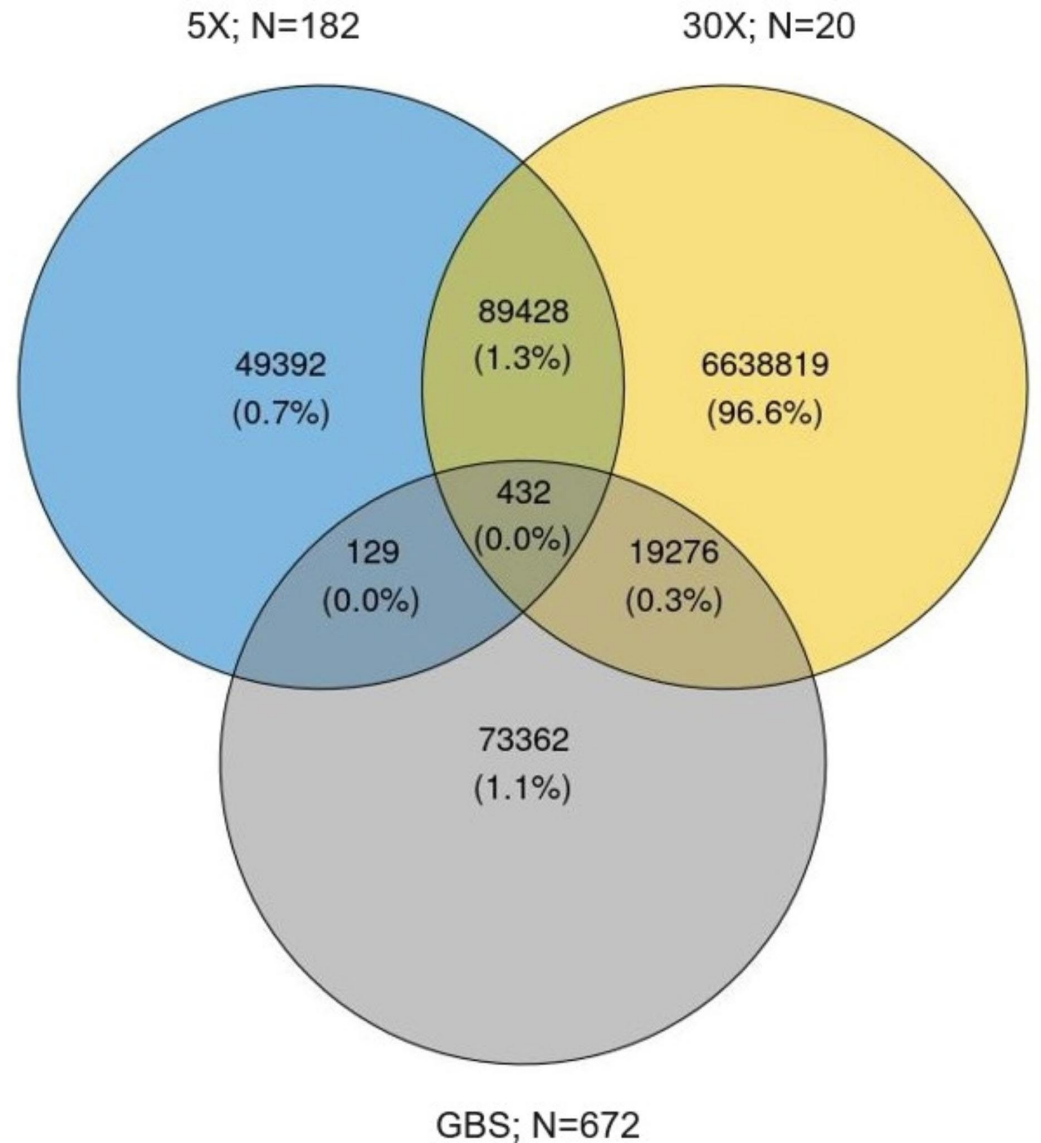
Venn diagram for the number of SNPs discovered in the East Canada group with each technology. 5X: whole genome sequencing with a depth of 5X; 30X: whole genome sequencing with a depth of 30X; GBS: Genotyping-By-Sequencing; N indicates the sample size

Using the same alignments with the genome assembly, two different approaches were deployed to detect SNPs from the GBS dataset which yielded different SNP numbers (Fig. 1). In the first approach, a total of 1,061,545 SNPs were detected using the platypus SNP caller (Table 1) which yielded a total of 93,199 SNPs (8.7%) when filtering for a minimum coverage to call a SNP. After filtering for a minimal MAF and maximal number of missing genotypes, 16,441 of these were retained for further analyses. In the second approach using the *stacks_workflow* approach, 6,050 high-quality SNPs were discovered after the removal of 873 linked SNPs and 1, 487 putatively duplicated SNPs (see supplementary material S1 for details at each step and stacks_workflow for reasoning). Increasing the minimum value of the MAF parameter reduced the final number of markers identified with this approach by about two-thirds, resulting in 2,580 high-quality SNPs that were polymorphic across boreal and migratory ecotypes. These sets of SNPs presented various degrees of overlap (Fig. 3).

### SNP selection for array design

Three sets of SNPs were included in the genotyping-chip array design: a first set of SNPs regularly distributed every 50 kb over the entire genome, a second one that allowed to assign a sample of unknown origin to a particular ecotype and genetic group from Eastern Canada, and a third set including SNPs related to adaptive behavioral variations. As the objective was to delineate an array of SNPs not restricted to particular parts of the species distribution, SNPs were first pooled according to geographic origin considerations to capture the largest genetic diversity and then the overlap between pools allowed to identify a promising SNP set usable in any caribou/reindeer genomic investigations.

Pooling both SNP sets found in the Northeastern genetic pool sequencing (high and low coverage) yielded a set of 6,798,838 unique SNPs while pooling SNPs found in Northwestern Canada and Eurasia yielded a set of 3,677,928 unique SNPs. Overlap between these two SNP sets resulted in a total of 1,723,567 SNPs widely spread over the species distribution in both North America and Eurasia. A custom in-house Python script selected 52,952 SNPs regularly distributed over the genome with a targeted distance of 50 kb between successive SNPs. As the genome assembly included scaffolds as small as 1 kb, a number of these were not represented by this SNP set. Nevertheless, these SNPs still represented 97.5% of the genome assembly, leaving only 65,939 kb not represented by the array. In addition, this SNP set included a total of 1,626 SNPs located upon the 22 largest scaffolds likely representing the caribou X chromosome according to alignments with the *Bos taurus* X chromosome (Supplemental Data S2).

**Fig. 4:**
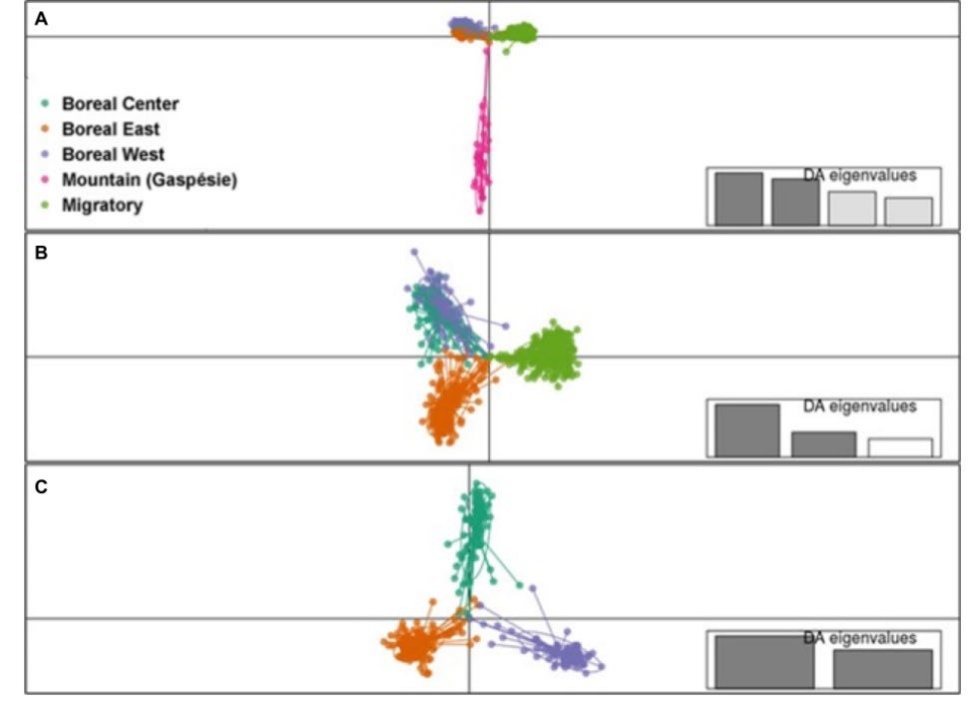
Discriminant analysis of principal components (DAPC) showing (A) Boreal, Mountain and Migratory ecotypes, (B) the three boreal and the migratory genetic groups and (C) the three boreal forest populations. Inset indicates the number and contribution of axes from principal component analysis retained in the DA.

The second panel aiming at ecotype and genetic group assignment in Eastern Canada was selected by applying a discriminat analysis of principal components (DAPC) to the set of 16,441 high quality SNPs found analyzing GBS data (Fig. 4). It revealed a hierarchical population structure with a first level differentiating the mountain ecotype, only composed in our sampling by one population with a small effective population size (Gaspésie, Fig. 4 A). Excluding this ecotype, a second DAPC showed a split between migratory and boreal (sedentary) caribou (Fig. 4B), while a third DAPC including only boreal samples showed a genetic divergence between the three boreal genetic groups of populations (namely West, Centre and East; Fig. 4 C). Selecting the SNPs presenting the highest loadings, 2,127 SNPs discriminated the mountain population from the other samples, 2,116 SNPs discriminated the migratory ecotype from the sedentary one, while 2,323 SNPs allowed to differentiate the three subpopulations of sedentary boreal caribou. A few SNPs overlapped between the three sets and a total of 6,235 SNPs were submitted for the array design. This SNP set was supplemented with the SNPs identified using the second GSB approach (N = 2,302), which included SNPs with lower MAF. Therefore, the assignment panel finally encompassed a total of 8,537 SNPs.

From the 2,256 behavior SNPs previously identified [22, 23], 1,877 SNPs presented flanking sequence mapping to a unique location in the genome assembly [21]. Among these, 1,336 (71%) were also found polymorphic in our sequenced samples. However, all 1,877 behavior SNPs were also submitted for array design as they may be of use in other populations than the ones sampled for the present study.

### Illumina array design

For each SNP of the three panels, 20-bp flanking sequences were analyzed for high variability (other SNPs occurrence). SNPs within windows of high variability were discarded according to Illumina’s guidelines. The first design was then tested by genotyping a total of 488 samples representing all populations from East Canada.

It was thus expected to find polymorphism for each SNPs from the genome-wide since only common SNPs were selected for this SNP set, for most SNPs from the ecotype-assignment SNP set as each population was represented, and 71% of the behavior SNPs in line with the proportion found in our sequencing data. This genotyping showed a subset of unsuccessful SNPs, leading to a second design with additional SNPs to replace those and that was also tested using a total of 1,333 samples.

#### *Initial desig*n

A total of 63,366 SNPs were provided to Illumina to design the first array. A conversion rate of 87% was obtained from this first version of the array, meaning that 87% of the SNPs provided for design were still part of the array at the end of the design process. The missing SNPs were composed of 6,864 from the genome-wide set, 803 SNPs from the assignment set, 286 SNPs from the GBS *stacks_workflow* set, and 230 from the behavior set. The average distance between the successfully designed SNPs was 48,353 bp but 7,554 gaps over 100,000 bp remained.

Testing the genotyping platform with 488 samples (including a number that also served for SNP discovery) showed a per-sample call rate of 94.8% and a reproducibility of 98.8%. In addition, testing the SNP-ship with those samples revealed the presence of uninformative SNPs; 2,506 of them had no call (missing genotypes for all samples) and 8,713 had no alternative allele (monomorphic) which was not the case in sequencing data. Analyzing raw data showed complex clustering of intensities and blasting the 50-bp flanking regions of these uninformative targets against the genome revealed a lack of specificity as a probable cause for the occurrence of these monomorphic and missing genotypes. Indeed, 50% of SNPs with no call (n = 1,269) and 70% of monomorphic SNPs (n = 6,087) presented immediate flanking sequences aligning to more than one region of the genome. Contrastingly, each successful SNP presented the same genotypes for samples that were both genotyped and sequenced with enough depth at the targeted nucleotide (N = 185), and presented 50 bp-flanking sequence blasting to a unique position in the genome assembly with a high similarity score. Given the availability of many more high-quality SNPs for replacement of these faulty probes, a second array design was done. For each uninformative SNP, the nearest potential replacement SNP in a − 20 kb to + 10 kb window was selected and SNPs that could not be replaced were submitted to re-design. Respectively, a total of 6,862 new candidate SNPs and 11,797 SNPs were submitted for this second-round design for a total of 18,659 newly submitted SNPs.

#### Second design

The second-round beads were added to the first bead pool; thus 76,050 SNPs were targeted in total, and the platform was tested using 1,333 samples. Data indicated 3,966 SNPs (2,509 of first design + 1,457 of second design) never returning any genotypes (= missing genotypes), as well as 8,749 SNPs (8,713 of first design + 36 of second design) presenting no alternative alleles (= monomorphic SNPs). Overall, this second design allowed to genotype the samples for a total of 63,336 validated probes with an average sample call rate of 97.8% and an average reproducibility of 98.7% (N = 7). Some redundancy in target loci that required additional design due to sequence complexity, i.e. occurrence other SNPs in the flanking sequences, led to 4,852 SNPs being detected by two probes. By sub-panels, these figures account for 53,580 probes for the genome-wide distribution (49,725 unique loci), 8,058 probes for the ecotype/genetic group assignment panel (representing 7,349 loci), 1,698 for the behaviour panel (representing 1,410 loci) and 7 probes for the SRY gene. Non-informative SNPs are listed in Supplemental Data S3 and can be removed from the array layout.

Overall, the spacing between successive SNPs was 44,646 bp (median = 48,973, std = 34,371). Linkage disequilibrium (LD) was estimated in six Eastern-Canadian genetic groups to determine if the SNP density was sufficient (Table 2).

**Table 2:** Average linkage disequilibrium within ecotypes and genetic groups from the Quebec province (Canada) for all SNPs.

Ecotype/genetic group	Average-LD
Mountain_Gaspésie	0.1710
Boreal_Western Québec	0.1099
Boreal_Central Québec	0.0996
Boreal_Eastern Québec	0.0397
Migratory_Rivière George	0.0579
Migratory_Rivière aux Feuilles	0.0260

List of Supplementary data

S1: Details for each step of the stacks workflow

S2: List of SNPs positioned on the X chromosome

S3: List of IDs of faulty probes that may yet be found in the resulting vcf

A very high density will likely lead to high LD estimates, meaning that neighbouring SNPs are generally inherited together and thus provide the same information. A very low LD value, by contrast, would be representative of insufficient coverage. As such, some LD is needed but extreme values are to be avoided. Estimates showed some level of LD in all populations and an expected increased value in the lowest effective-size population (Gaspésie), indicative of relatively limited genetic diversity, whereas the lowest value was observed in the large migratory herd (Rivière-aux-Feuilles).

### Sexing

Various sampling strategies, including non-invasive opportunistic sampling from feces for instance, result in samples with no additional information regarding the individual. In such cases, the sample sex is unknown although being important information for conservation purposes. We therefore developed two subsets of SNPs to allow sexing samples from genotypes: (1) a set of SNPs located on the X chromosome that should present homozygosity in males and various degrees of heterozygosity in females and (2) a set of SNPs located in the SRY gene (specific to the Y chromosome) that should yield a homozygous genotype in males and missing genotypes (no call) in females.

**Fig. 5:**
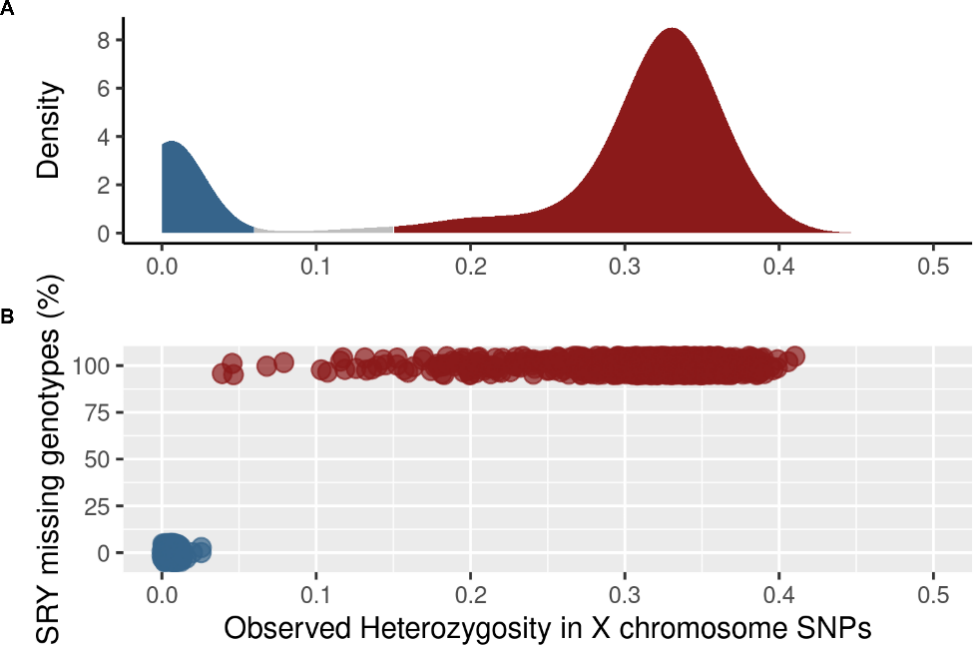
Sexing samples using the SNP chip. (A) Distribution of observed heterozygosity over the 1438 SNPs from the X chromosome; the left peak (max = 0.005) represents the homozygous genotypes from males (in blue) while the right peak (max = 0.33) represents the more heterozygous genotypes from females (in red). (B) Combining the extent of the chromosome X heterozygosity with call-rates of the SNPs in the SRY gene (absent from female genome); the cluster on the bottom left represents the males where a positive call was generated for all 6 probes while the upper cluster represents the females with complete missing genotypes

The successful SNP set included 1,626 SNPs likely located on the X chromosome. As such, males should be monomorphic across this subset of SNPs except for those possibly located in the pseudoautosomal chromosomal regions where sequence similarity may allow for successful hybridization on both Y and X chromosomes. Only confirmed monomorphic SNPs in males were kept for further analyses. The average observed heterozygosity (Ho) per individual estimated from the remaining chromosome-X 909 SNPs revealed an expected bi-modal distribution with an inflection point at 0.08 (Fig. 5 A). The narrow mode with a maximum number of samples at 0.01 represented confirmed males while the larger one culminating at 0.33 represented confirmed females. In addition, 7 probes within SRY gene sequence were successfully designed from the 8 possible coordinates we submitted to the Illumina team. As one probe didn’t yield any genotype for any sex, the final design included 6 functional probes detecting the presence of the SRY gene located on the Y chromosome in males and yielding no-call in females. Both methods were in perfect agreement when considering thresholds of 0.03 for chromosome X Ho and 50% for missing genotypes in SRY SNPs to distinguish males from females (Fig. 5B). From field metadata, the sex was known for 1,228 individuals and all were confirmed by these genotypes.

## Discussion

An Illumina bead chip for caribou and reindeer (*Rangifer tarandus*) was developed using three different sequencing strategies and samples covering populations distributed world-wide. We believe the availability of this new tool will facilitate the management and conservation of the whole species. As described by Yannic et al. [18], the species split into two main genetic clusters, the Euro-Beringia/Western North American and Northeastern American ones, which were both sampled in our work. The importance of sampling both clusters was exemplified with the limited overlap between the SNP catalog from Quebec/Eastern Canada compared to the one combining samples from Western Canada, Alaska and Eurasia. We selected SNPs for the genome-wide panel within this overlap of common SNPs across populations to ensure the suitability of the genotyping platform for any caribou or reindeer populations worldwide. It can be assumed that the vast majority of these SNPs showing allelic variance worldwide are neutral, not under selection. This is a positive feature in the sense that allelic frequencies are not expected to shift rapidly or become fixed over time. Consequently, the panel is expected to remain informative for a long period of time.

The Platypus variant caller was used for SNP discovery in this study since it has been reported to have one of the highest accuracies [24] and we additionally selected the SNPs with the highest quality. However, this is achieved at the expense of sensitivity and Platypus likely misses a number of variants that actually exist [24]. Therefore, other variants are likely occurring in the populations we investigated but we prioritized robustness of the found SNP sets rather than finding all possible SNPs since the objective was to integrate these into the genotyping chip design.

In order to offer a genome-wide perspective, SNP density and uniform genome distribution are important when designing a genotyping array. The genome-wide panel targets 49,725 SNPs within a 2.59 Gb genome assembly [21]. The average spacing between successive SNPs is 44,646 bp and is comparable to other commercial SNP arrays developed for livestock such as the BovineSNP50 [11], the PorcineSNP60 [10] and the GoatSNP50 [25]. These SNPs chips with a uniform genome-wide distribution are useful for genome-wide association studies, parentage analyses [12, 26–30], or population size and inbreeding estimation [31]. While higher density panels may increase accuracy of genomic estimates, it has been shown that the closely located markers are more often in complete Linkage Disequilibrium (LD) therefore offering the same information as the neighbouring SNPs with no specific added value [32, 33]. As such, our 63,336 SNP panel likely offers a good balance accounting for an efficient genomic coverage without extensive duplicated information due to LD and genomic analyses relying on this SNP chip should be highly accurate.

A SNP panel destined for ecotype and genetic group assignment does not need full coverage of the genome. A low number of SNPs is sufficient, provided that the selected SNPs are highly informative [34]. It has been demonstrated by previous forensics studies that only a few tens or hundreds of SNPs are needed for assignment with certainty to population of origin [35, 36]. However, using SNPs representing the present-day population’s structure might not be suitable to assign individuals years from now since allele frequencies can change over time due to genetic drift, selection, or gene flow [37, 38]. Therefore, the 8,058 SNPs selected to discriminate the three ecotypes found in Eastern Canada represent a very comprehensive panel that is expected to remain accurate through time.

Selecting SNPs for the panel discriminating ecotypes in Eastern Canada was done using a DAPC. As described by Jombart et al. [39], PCA will detect the variability between the groups, but also within them whereas DAPC concentrates on the discrimination of individuals from the established groups. It was expected that the cluster of individuals from the Gaspésie population (mountain ecotype) would be further away from the other clusters since these individuals are genetically diverging owing to population isolation [18, 40]. Indeed, the Gaspésie population is the only one south of the St. Lawrence River, thus isolated from the major part of the caribou distribution. When Gaspésie individuals are pulled out of the analysis, DAPC can better discriminate the genetic diversity between the boreal forest and the migratory ecotypes. Within the boreal ecotype, the lower variability between the boreal center and boreal west groups described in Yannic et al. [18] has also been detected using SNPs as the two clusters were superimposed (Fig. 4).

In addition to genetic metrics, the genotyping platform allows to determine the animal’s sex using SNPs located on the X-chromosome. This is instrumental when sampling was non-invasive or conducted without recording the information (e.g. archived or pellet samples). The analysis was based on the extent of heterozygosity where females should display some heterozygous genotypes whereas males will always display all homozygous genotypes since bearing only one X-chromosome. Sexing using this approach proved to be highly efficient where all animals were appropriately sexed. Some females from populations with very limited effective size and known to be under increasing inbreeding depression, displayed very low diversity on the X-chromosome. The platform has another means of sexing by detection of the SRY gene only located on the Y-chromosome. The bead pool contains 6 fully functional probes targeting SRY. Detection of the presence of the gene is based on the generation of sufficient signal over background. GenomeStudio, which is Illumina’s software for genotype calling, is not designed for handling monomorphic (because it is not a SNP) haploid (because it is only on the Y-chromosome) signals. With modified settings, samples resulting in positive signals or no-calls for all 6 probes were all associated with the correct known gender. As such, the animal sex can be confirmed by the two complementary approaches.

## Conclusion

Overall, we report here the design of the first commercially available SNP chip for the landmark species *Rangifer tarandus*, available at the Centre d’expertise et de services de Génome Québec (Montréal, Canada; https://cesgq.com). The platform has been designed to be efficient for all caribou and reindeer populations, and tested with a large number of samples to ensure its robustness. Given the number of SNPs targeted by the platform (63 K), it should prove to be instrumental for many aspects of caribou and reindeer management and protection.

## Methods

### Biological material, DNA extraction and sequencing

Our sampling for WGS purposes included three subsets of individuals: (1) a group of reindeer (N = 10) from multiple geographical locations in Eurasia (Svalbard in Norway; Yamal, Lena-Olenyk, Wrangel, and Nenetsky in Russia), (2) a group of caribou from western Canadian populations (N = 10), and (3) a large set of samples from eastern Canadian caribou populations (N = 182) (Fig. 1). Aside from the eastern Canadian caribou populations representing three ecotypes (migratory, boreal and mountain) of *Rangifer tarandus caribou*, Canadian sampling included three migratory barren-ground caribou (*R.t. groenlandicus*) populations (Beverly, Bathurst, and Bluenose East, NWT, Canada), two western caribou (*R.t. caribou*) populations (Besa Prophet, BC; The Bog, MB, Canada) representing the boreal ecotype and one migratory Barren-ground x Peary caribou (*R. t. groenlandicus* × *pearyi*) population (Dolphin-Union, NWT, Canada).

Sampling for GBS purposes included 672 individuals (Fig. 1) from various eastern Canada caribou populations also representative of three caribou ecotypes: the migratory, the boreal and the mountain caribou (Fig. 2). These populations sampled for GBS included 278 migratory caribou, 29 mountain caribou from Gaspesie and 365 boreal caribou from three previously identified genetic groups: Boreal East (n = 182), Boreal Center (n = 89) and Boreal West (n = 94) [19]. For each individual, the ecotype assignment at capture was ensured by previous telemetry tracking performed by the provincial services in charge of caribou populations monitoring.

Genomic DNA was extracted from ear punches and hair follicles (Table 1) using the DNeasy Blood and Tissue kit (QIAgen, Toronto ON, Canada) following manufacturer’s recommendation. Evaluation of the DNA quantity and integrity was performed using genomic DNA screentape on a 4200 TapeStation system (Agilent, Santa Clara, CA, USA). Whole genome sequencing libraries were constructed at Centre d’expertise et de services de Génome Québec (Montréal, QC, Canada) using a shotgun DNA library preparation strategy (NEBNext Ultra II DNA Library Prep Kit, New England Biolabs, Whitby, ON, Canada). GBS library preparation was performed at the *Institut de Biologie Intégrative et des Systèmes* (IBIS, Université Laval, Québec, QC, Canada). Briefly, two-enzymes (PstI and MspI) were used to digest genomic DNA followed by ligation of a unique barcode index for each sample. Both WGS and GBS libraries were sequenced at the Centre d’expertise et de services de Génome Québec (Montréal, QC, Canada). WGS and GBS samples were sequenced aiming at an average coverage of 5X. In addition, we relied on high coverage WGS data for 20 individuals from the Quebec province (Canada) that were previously used to detect copy number variations and published in 2022 [21]. Raw reads from these samples were also retrieved and used for SNP detection.

### Detection of high-quality SNPs in WGS and GBS

#### SNP detection in WGS

All WGS reads were checked for sequencing quality using FastQC (Andrews, 2010), trimmed using Trimmomatic 0.36 [41], and aligned using BWA_0.7.17 [42] to the latest ULRtarCaribou_2 reference (GCA_019903745.1; [21]) made of 13,994 scaffolds representing 2.59 Gb (Fig. 1). The genome was first repeat masked using REPEATMASKER_4-0-7 and then, genotypes were called using Platypus 0.8.1 [43] (Fig. 1) with a minimum mapping quality of 30, a maximum number of 3 variants per 100 bp windows, and a per-individual minimum of 3 reads with satisfying mapping quality to call variant, except for the high coverage WGS where the minimum of 12 reads with satisfying quality was required.

#### Variant filtering for high quality SNPs in WGS

False positives are a well-known issue when detecting polymorphisms; thus, a number of methods have been deployed to circumvent this problem. Among those, repeatedly finding a polymorphism among several individuals is a robust way to ensure the existence of a SNP. In addition, rare alleles occurring in only a few individuals and likely limited to one population were avoided, as the objective was to design a genotyping-chip with the largest usability. Thus, we filtered variants identified from WGS for only bi-allelic variants (= SNPs) and a minimum minor allele frequency exceeding 20%. This threshold translated into a minimum of 4 minor alleles in the small groups of 10 individuals (Eurasian and Canadian groups) and a minimum of 72 alleles in the large sample set from the Quebec province. In addition, we discarded SNPs presenting an observed heterozygosity over all samples exceeding 0.49 (indicating that every sample is heterozygous) as systematic heterozygosity over large sample sets is likely indicative of paralogs and not SNPs.

Given the low coverage (5X) for this WGS, we can expect a few individuals to not present reads (or not enough of those) in some genome regions, resulting in missing genotypes for a particular locus. Contrastingly, a SNP with a lot of missing genotypes may indicate that the SNP occurs in a complex region impeding genotyping which should be avoided in order to maximize the success rate at probe design. Thus, a maximum of 30% of missing genotypes was tolerated in the large Quebec populations group while a 50% threshold was applied for the two other groups since fewer individuals were involved (Fig. 1). Finally, only 10% of missing genotypes were tolerated in the high coverage data given that genome regions randomly missing reads with a 30X coverage sequencing should be rare (Fig. 1).

#### SNP detection and variant filtering in GBS

GBS data were obtained for a total of 672 individuals distributed over 3 ecotypes and 5 genetic groups from Eastern Canada named “Migratory”, “Gaspésie” (= mountain), “Boreal East”, “Boreal Center” and “Boreal West” groups. Two different approaches were deployed to detect SNPs from the GBS data. While one of the procedures aimed at identifying the SNPs most likely polymorphic in populations from many parts of the species distribution, the other aimed at identifying all SNPs from the sample set, thus more likely representative of the entire allele frequency spectrum, including rare SNPs (MAF < 1%).

In the first approach, SNPs were detected following the steps implemented in the FastGBS pipeline [44]. In this procedure, reads were mapped onto the recent genome assembly [21] using BWA [42] after demultiplexing with Sabre (https://github.com/najoshi/sabre) and cleaning using Cutadapt [45]. Then, SNPs were detected using the Platypus variant caller [43] with the parameters optimized for Illumina sequencing in FastGBS [44]. Subsequently, variants were filtered according to quality criteria including a maximum of two alleles of one nucleotide per variant (i.e. only biallelic SNPs), a maximum number of 3 SNPs per 100 nucleotides windows, and a minimum of 10 reads supporting the alternative allele. Finally, SNPs were filtered according to the following criteria: a maximum of two alleles (SNPs), a maximum heterozygosity of 0.49 (systematic heterozygosity being indicative of possible paralogy), a minimum of 70% of successfully genotyped samples, and a minor allele frequency (maf) > 0.2.

From the previously aligned BAM files of the 672 individuals that were sequenced in the GBS runs, a second method for SNP discovery was used. This second GBS pipeline included steps implemented in STACKS v2.40 and custom script from stacks_workflow (https://github.com/enormandeau/stacks_workflow) to filter for high-quality SNPs according to different criteria. In this approach, population parameters were used to filter out potentially low-quality samples and SNPs. Before running the pipeline, we excluded samples from the Gaspésie population and samples that presented BAM files smaller than 20 MB. The former to maximize a SNP discovery focused on polymorphism among caribou from the migratory and boreal ecotypes, and the latter as they would not pass the minimum read coverage threshold along the following steps because of too little alignment data and only impeded early steps of SNP cataloging. The *gstacks* module was executed to assemble and merge paired-end contigs, call variant sites in the populations and genotypes in each sample. Then, the SNP set was filtered for SNPs presenting at least 60% of genotypes per population (-r 0.6), a minimal allele coverage of 4X and a minimum of two samples presenting the rare allele (-p 2). Individuals with less than 40% of missing genotypes showing normal (no outlier) values of relatedness and heterozygosity were kept. We also investigated SNP “anomalies” based on McKinney, Waples, Seeb and Seeb [46], discriminated SNPs found in duplicated loci and discarded linked loci (in the same region and presenting matching genotypes). Finally, in order to constrain the SNP discovery to a high-quality, high-confidence, and powerful panel to discriminate caribou ecotypes, we filtered by increasing the minimum number of samples presenting rare alleles to 23, which brings further confidence that a rare allele will not be specific to one individual but likely present in future population sampling (proxy for MAF between 0.01 and 0.02).

### SNP selection for the array design

#### Whole genome coverage

In order to select SNPs that would be found worldwide and not restricted to one region of the species distribution, SNPs found in East-Canada (low coverage and high coverage WGS), where sampling effort was more extensive, were pooled together, while SNPs found in the other groups (West-Canada and Eurasia) were grouped into another SNP set. An overlap between the two SNPs pools made sure that SNPs would be detectable in any population worldwide. The flanking 250-mer sequences of the selected SNPs overlapping both sources were then aligned to the genome assembly using Mummer 4.0.0 [47] and those with flanking sequences mapping to a unique location in the genome were added to the list as candidates for chip design. An in-house Python (3.6) script was then developed to select SNPs evenly distributed on the caribou genome in order to have maximum coverage. The targeted average distance between each SNP was 50 kb, yielding a total of approximately 50,000 SNPs; this distance could be as high as 60 kb or as low as 30 kb.

#### Ecotype and genetic group assignment for northeastern samples

SNPs derived from GBS data were used to delineate a candidate SNP set that would allow the assignment of a sample to its genetic group of origin in the Quebec province. The Quebec sample set was analyzed using a discriminant analysis of principal components (DAPC) implemented in the “adegenet” package [48] from R (R Core Team, 2021) to find SNPs maximizing the genetic differentiation between the groups of individuals (migratory, mountain, western boreal, center boreal, eastern boreal). The *loadingplot* function was used to get the most contributing SNPs and a total of 6,000 were selected to ensure a high discriminating power. Given the hierarchical population structure (see results), three DAPCs were performed, each analyzing a different level of population structure.

#### Behavioural SNPs

A set of 2,256 SNPs related to migratory behavior or associated with geographic or environmental variations was identified in caribou from western North America [22, 23]. These SNPs of interest were included in the array design. The flanking sequences of these SNPs found using a *de novo* GBS approach were aligned to the genome and those mapping a single unique location with no other SNPs in the surrounding 20 bp were added to chip design.

### Sexing samples using the SNP-chip

The first sexing strategy was based on SNPs located on the X chromosome which should present higher heterozygosity in females than in males that should appear homozygous (haploid; only one X chromosome) for each of those SNPs. Scaffolds of the caribou genome assembly have been previously compared to the bovine genome [21] and large scaffolds matching the X chromosome were identified. For each sample, we measured the average observed heterozygosity over the SNPs located within these scaffolds expecting to identify clusters of samples with either low heterozygosity (males) or medium to high heterozygosity (females).

As the possibility of females with low genetic diversity appearing as males could not be entirely discarded, a second approach using the SRY gene was developed to ensure proper sexing despite the high number of SNPs located on the X chromosome and successfully included on the chip. As the SRY gene is located upon the Y chromosome and thus specific to males, probes targeting this gene interrogated using a SNP-chip should yield apparent homozygous genotypes in male samples while showing missing alleles in females. To design probes for the *Rangifer tarandus* SRY gene, short-read data from the male sample with the highest sequencing yield in the set of 30X-WGS samples was used to assemble a male genome using the DISCOVAR-DENOVO bioinformatic tool (*DISCOVAR: Assemble Genomes, Find Variants.*, n.d.). This assembly process resulted in a highly fragmented genome (N50 = 21,090 bp). The publicly available bovine SRY gene sequence (https://www.ncbi.nlm.nih.gov/gene/280931) was then used as bait to find the contig harboring the orthologous SRY gene. In our new male assembly, the complete gene sequence (690 bp) was found within a single contig. A total of 8 nucleic positions separated by 100 bp were chosen to allow the design of non-overlapping Illumina probes to be included in the SNP-chip.

### Illumina array design

SNPs neighboring other SNPs within the flanking 20 bp windows (even those filtered out according to quality criteria such as low MAF for instance) were discarded to avoid interference with probe hybridization. The candidate SNPs were then provided to *Illumina concierge* as 101-mer nucleotide sequences with the SNP and alleles showed at position 51.

This first array design was tested using 488 samples, including the 20 individuals with high coverage WGS, that were hybridized at the Centre d’expertise et de services Génome Québec (Montréal, QC, Canada). Raw data were analyzed using the GenomeStudio informatic tool and genotypes were inferred from clusters of hybridization intensity ratios. The call rate and the number of SNPs with no alternative allele were computed from this first data set. The first results revealed a subset of unsuccessful SNPs (see results) that were replaced within a new Illumina’s array design that was also tested using 1,333 samples hybridized and genotyped at the same service platform.

## Electronic supplementary material

Below is the link to the electronic supplementary material.

Additional file 1: Details for each step of the stacks workflow

Additional file 2: List of SNPs positioned on the X chromosome

Additional file 3: List of IDs of faulty probes that may yet be found in the resulting vcf

## Data Availability

(ADM) The datasets generated and/or analysed during the current study are available in the NCBI SRA repository, https://www.ncbi.nlm.nih.gov/bioproject/PRJNA846266.
